# A GC–MS method for the determination of furanylfentanyl and ocfentanil in whole blood with full validation

**DOI:** 10.1007/s11419-018-0449-2

**Published:** 2018-10-22

**Authors:** Nektaria Misailidi, Sotiris Athanaselis, Panagiota Nikolaou, Maria Katselou, Yannis Dotsikas, Chara Spiliopoulou, Ioannis Papoutsis

**Affiliations:** 1 Department of Forensic Medicine and Toxicology, Faculty of Medicine, National and Kapodistrian University of Athens, 75 Mikras Asias, 115 27 Athens, Greece; 2 Laboratory of Pharmaceutical Analysis, Department of Pharmacy, National and Kapodistrian University of Athens, 157 71 Athens, Greece

**Keywords:** Furanylfentanyl, Ocfentanil, Acetylfentanyl, Butyrfentanyl, Whole blood, GC–MS

## Abstract

**Purpose:**

Fentanyl analogues are popular in recent years among drug addicts and have been related to many overdoses and deaths worldwide. Furanylfentanyl, ocfentanil, acetylfentanyl and butyrfentanyl are among the most common of these drugs. Methods for the determination of furanylfentanyl and ocfentanil by gas chromatography–mass spectrometry (GC–MS) in biological samples do not exist, and therefore, their development would be extremely useful for routine toxicological analysis.

**Methods:**

A GC–MS method was developed and fully validated for the determination of furanylfentanyl and ocfentanil in whole blood. This method was also suitable for the determination of acetylfentanyl and butyrfentanyl. The method included solid-phase extraction after protein precipitation using acetonitrile, and it was applied during the toxicological investigation of forensic cases. Methadone-*d*_3_ was used as internal standard for the quantification of the analytes.

**Results:**

The limit of detection and limit of quantification values were 0.30 and 1.0 ng/mL for furanylfentanyl and ocfentanil and 0.15 and 0.50 ng/mL for acetylfentanyl and butyrfentanyl, respectively. The calibration curves were linear (*R*^2^ ≥ 0.993) from 1.00 to 100 ng/mL for furanylfentanyl and ocfentanil and from 0.50 to 50.0 ng/mL for acetylfentanyl and butyrfentanyl. The recoveries were not lower than 85%, while accuracies and precisions were not greater than 6.0% (% error) and 8.0% (% relative standard deviation), respectively, for all four fentanyl analogues.

**Conclusions:**

The developed method is the first one in the literature for the detection of furanylfentanyl and ocfentanil in biological fluids by GC–MS, and it provides very high sensitivity comparable to that by liquid chromatography–tandem mass spectrometry.

## Introduction

Fentanyl analogues were initially developed to find opioid analgesics with better therapeutic indices, in terms of potency, than fentanyl. Many of the fentanyl analogues are considered to be novel psychoactive substances (NPS) [1–3]. During the 1970s fentanyl and its analogues were detected for the first time on the illicit drug market. Approximately 1400 fentanyl analogues had been synthesized and more than 200 of them had been described in the literature in toxicological or pharmacological studies until 2004 [4], but only three of them have been approved for medical use. In recent years, misuse of fentanyl and often even more potent fentanyl analogues (fentanils) has become more prevalent; the market has increased in complexity and harms have increased [3]. Following this outbreak, local authorities have placed many fentanyl analogues under control in order to limit their use [5–8]. As a result, many national governments have recognized most notably China as the source of the vast majority of the fentanyl and fentanils produced for the illicit drugs market, and international organizations have placed all or some of the fentanils under legislative control in an attempt to control their production and trade and to limit their use. In response to the legislative controls of specifically named substances, especially where generic legislation based on alterations to the fentanyl structure is not in place, newer fentanils continue to appear [3]. The development and validation of analytical methods for the determination of such substances are required for the analysis of biological samples for clinical and forensic toxicological purposes.

Four of the most recent fentanils appearing on the illicit drugs market are furanylfentanyl, ocfentanil, acetylfentanyl and butyrfentanyl. Only a few analytical methods validated for the determination of these fentanyl analogues have been published. Immunoassays generally used for screening of urine samples for fentanyl cannot differentiate fentanyl analogues from one another or from fentanyl [9–13]. For the confirmation of these four fentanyl analogues separately in blood [14–24], urine [14–16, 18, 20, 22, 24–29], vitreous humor [14, 15, 18, 20], bile [18] and other matrices [14–16, 18], mainly liquid chromatography (LC) was employed [18–24, 26–28]. Gas chromatography–mass spectrometry (GC–MS) has also been used for the determination of some fentanyl analogues in biological fluids [14–17, 25].

To our knowledge, there is no published validated method for the determination of furanylfentanyl and ocfentanil in biological specimens by GC–MS. The aim of this study was the development and validation of a fast, sensitive and specific GC–MS method for the determination of these two fentanils in whole blood. This method was also used for the determination of acetylfentanyl and butyrfentanyl in the same matrix. The GC–MS instrument is most widespread in forensic toxicological laboratories in the world, and it is most fundamental as a reliable instrumental analysis. Therefore, the establishment of quantitative analysis of circulating illicit drugs, such as fentanils, by GC–MS is essential.

## Materials and methods

### Materials

Furanylfentanyl hydrochloride (powder ≥ 98%), ocfentanil hydrochloride (powder ≥ 98%), acetylfentanyl hydrochloride (powder ≥ 98%) and butyrfentanyl hydrochloride (powder ≥ 98%) were purchased from Cayman Chemical Company (Ann Arbor, MI, USA), while methadone-*d*_3_ (methanolic solution 1.0 mg/mL, ≥ 99.9%) was purchased from LGC Promochem (Molsheim, France). Other drugs and their metabolites used were of the highest purity commercially available.

The solvents used (acetonitrile, dichloromethane, ethyl acetate, hexane, isopropanol, ammonium hydroxide) were of high-performance liquid chromatography (HPLC) grade and were purchased from Merck (Darmstadt, Germany). Bond Elut Certify, Bond Elut Certify II and HF Bond Elut C18 solid-phase extraction (SPE) cartridges were obtained from Agilent Technologies (Santa Clara, CA, USA).

### Preparation of standard solutions, calibrators and quality control samples

A standard stock solution (at a concentration of 10 μg/mL) of each of the four analytes was used for the preparation of three calibrator working solutions. The first calibrator working solution was at a concentration of 0.050 μg/mL for furanylfentanyl and ocfentanil and at a concentration of 0.025 μg/mL for acetylfentanyl and butyrfentanyl; the respective concentrations of the second calibrator were 0.2 and 0.1 μg/mL and for the third calibrator were 1.0 and 0.5 μg/mL, respectively. Pooled drug-free blood samples were spiked with the appropriate volume of the above working solutions giving calibrators at the concentrations of 1.0, 2.0, 6.0, 20.0, 40.0 and 100 ng/mL for furanylfentanyl and ocfentanil, and of 0.5, 1.0, 3.0, 10.0, 20.0 and 50.0 ng/mL for acetylfentanyl and butyrfentanyl. Two different working solutions were prepared for the preparation of the quality control (QC) samples. The first solution was at a concentration of 0.2 μg/mL for furanylfentanyl and ocfentanil and at a concentration of 0.1 μg/mL for acetylfentanyl and butyrfentanyl, and the respective second calibrator were 1.0 and 0.5 μg/mL. The concentrations of the three QC levels were 3.0, 30.0 and 80.0 ng/mL, for furanylfentanyl and ocfentanil and 1.5, 15.0 and 40.0 ng/mL for acetylfentanyl and butyrfentanyl. Finally, a working internal standard solution of methadone-*d*_3_, was prepared at a concentration of 2.0 μg/mL by dilution of the stock solution (1.0 mg/mL) in methanol.

### Sample preparation

The method included the combination of protein precipitation with acetonitrile and SPE of whole blood samples followed by GC–MS analysis. A volume of 50 μL of the working internal standard solution (methadone-*d*_3_, 2.0 μg/mL) was added to 1.0 mL of each whole blood sample. The blood samples were vortexed for 30 s. A 2-mL volume of acetonitrile was added to the samples, during the vortexing for protein precipitation. The samples were centrifuged at 3000 rpm for 5 min and the supernatant was collected. A volume of 3 mL of 0.1 M phosphate buffer (pH 6.0) was added to the supernatant and the samples were centrifuged at 3000 rpm for 5 min. Bond Elut Certify SPE cartridges were conditioned with 3 mL methanol, 3 mL deionized water and 1 mL of 0.1 M phosphate buffer (pH 6.0). The supernatant sample was loaded onto the cartridges with a flow rate of 1.0 mL/min. The columns were then washed with 3 mL of deionized water, 1 mL of 0.1 M phosphate buffer (pH 4.0) and 3 mL methanol, and dried under high vacuum (≥ 10 mmHg) for 10 min. Finally, the analytes were eluted twice with 2.0 mL of a freshly prepared mixture of dichloromethane/isopropanol/ammonium hydroxide (85:15:2, v/v/v). The eluents were collected, evaporated to dryness under a gentle stream of N_2_ at 40 °C and then reconstituted in 50 μL of ethyl acetate before GC–MS analysis. A volume of 1 μL was injected into the GC–MS system.

### GC–MS instrument and its conditions

A Shimadzu model GC-2010 equipped with a Shimadzu AOC-20i autosampler system and interfaced with a Shimadzu QP 2010S mass spectrometer (Shimadzu, Kyoto, Japan) was used in this study. A DB-5MS column (30 m × 0.25 mm i.d., 0.25 μm film thickness) was supplied by Agilent Technologies and used for the chromatographic analysis. Helium was used as the carrier gas at a flow rate of 1.0 mL/min. The GC–MS system was operating at the following conditions: initial column temperature at 100 °C, held for 1 min, increased at a rate of 30 °C/min to 300 °C and held for 5 min. The MS was operated in electron ionization (EI) and selected ion monitoring (SIM) modes. The three ions used for each fentanyl analogue were *m/z***283**, 240, 158 for furanylfentanyl, *m/z***279**, 176, 236 for ocfentanil, *m/z***231**, 146, 188 for acetylfentanyl, *m/z***259**, 146, 189 for butyrfentanyl, and *m/z***297**, 72, 161 for methadone-*d*_3_. The bold marked ions were used for the quantification of fentanils. Acetylfentanyl was eluted at 9.34 min, butyrfentanyl at 9.92 min, ocfentanil at 10.03 min, furanylfentanyl at 11.76 min and methadone-*d*_3_ at 7.461 min.

## Results and discussion

### Method development

The GC–MS method for the determination of furanylfentanyl and ocfentanil in whole blood was developed and validated. This method was also suitable for the determination of acetylfentanyl and butyrfentanyl in the same matrix. This method can be probably applied to whole blood samples in any forensic case involving these substances. It should be mentioned that fentanils are generally well suited for GC–MS analysis without derivatization.

The four fentanyl analogues were initially injected (50 μL of a combined working standard at concentration 1.0 μg/mL) into the chromatographic system in scan mode (*m/z* 50.0–500) using EI. The three most abundant and selective ions of the respective mass spectrum of each analyte were used as qualifier and quantifier ions, and the most abundant ions were used for the quantification of the fentanils.

The developed method includes sample pretreatment with protein precipitation using acetonitrile and SPE extraction with Bond Elut Certify columns. In the methods previously published, liquid-liquid extraction (LLE) was mostly used as the extraction method [13–15, 22]. LLE was tested during the optimization of the extraction method. Different pH values (7.0, 8.0, 9.0, and 10.0) were tested, as well as different organic solvents mixtures. These solvents were ethyl acetate, hexane/ethyl acetate (70:30, v/v), hexane/ethyl acetate/isopropanol (49:49:2, v/v/v), hexane/isopropanol (98:2, v/v), dichloromethane/isopropanol (90:10, v/v), dichloromethane/hexane/isopropanol (49:49:2, v/v/v), and dichloromethane/ethyl acetate (20:80, v/v). In all cases, there were interferences by endogenous matrix compounds. During the development of the extraction method, different SPE columns (Bond Elut Certify, Bond Elut Certify II, and HF Bond Elut C18) were also tested. A strong matrix effect was observed at the retention time of furanylfentanyl when Bond Elut Certify II and HF Bond Elut C18 columns were used. The hydrophobic and cation exchange properties of the Bond Elut Certify column made them suitable for the analysis of basic drugs, such as the studied fentanyl analogues. When the SPE column was tested, high recovery values and low matrix interference were observed (85% for all analytes), thus SPE extraction with Bond Elut Certify columns was chosen as the optimum extraction technique.

### Method validation

**Fig. 1:**
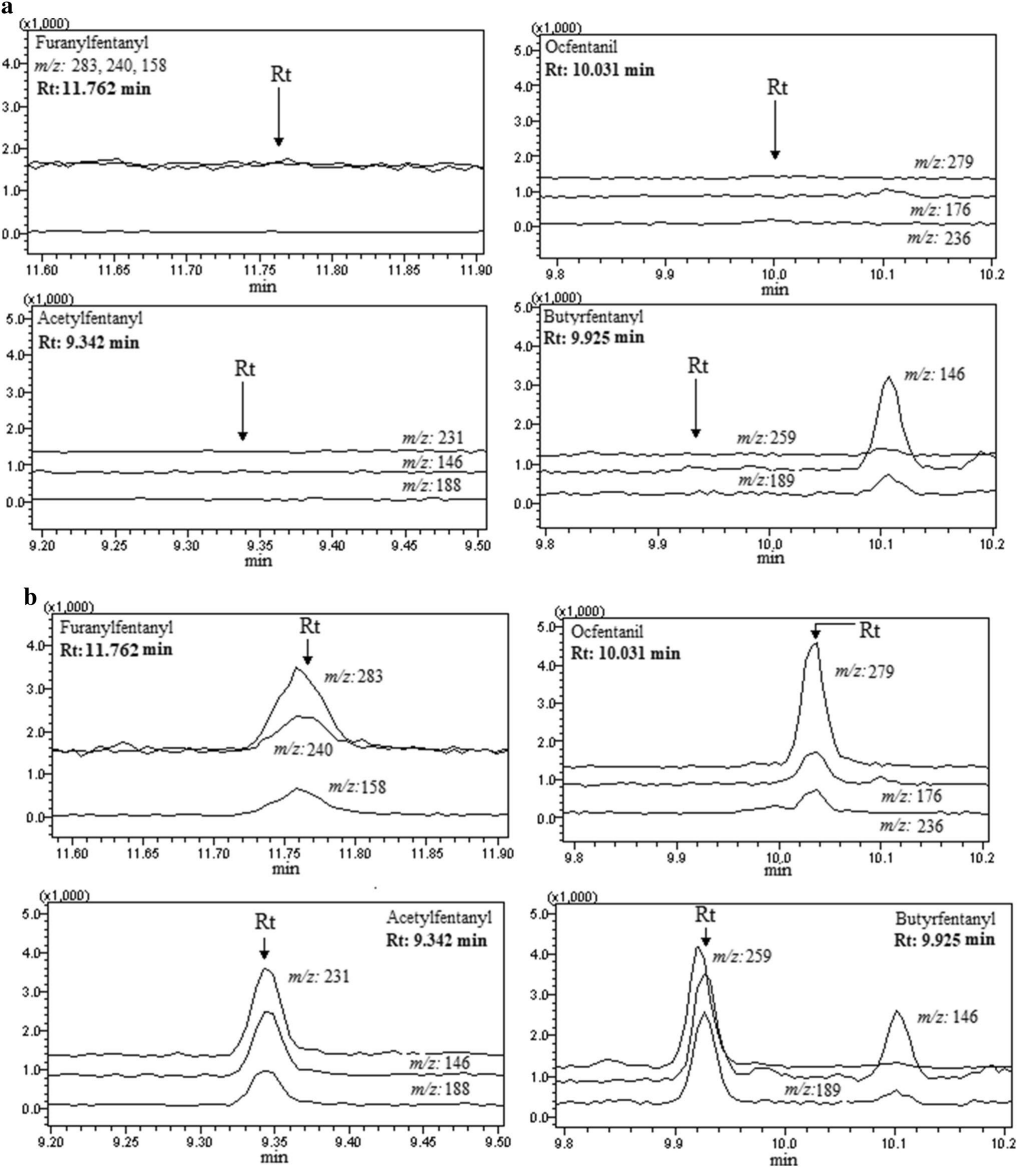
Representative selected ion monitoring chromatograms of: **a** a blank blood sample and **b** a spiked blood sample with the four fentanyl analogues at the respective limit of quantification (LOQ) concentrations (1.00 ng/mL for furanylfentanyl and ocfentanil, and 0.50 ng/mL for acetylfentanyl and butyrfentanyl). *Rt* retention time

The validation of the developed GC–MS method was performed according to international guidelines [30–32]. The following criteria were used to evaluate the GC–MS method: selectivity, specificity, limits of detection (LODs), limits of quantification (LOQs), linearity, absolute recovery, accuracy, precision and stability of spiked samples. The validation process was performed during four different days. Selectivity was estimated via analysis of six blank blood samples. After the matrix effect assessment in the selectivity study, negligible matrix interferences, from endogenous blood compounds, were observed at the retention times of all analytes of interest. Representative SIM chromatograms of a blank sample for all analytes are presented in Fig. 1a.

Specificity was examined by analysis of six spiked blood samples with illicit and medicinal drugs and their metabolites that are commonly detected in casework such as morphine, codeine, 6-acetylmorphine, methadone, buprenorphine, norbuprenorphine, fentanyl, norfentanyl, cocaine, benzoylecgonine, ecgonine methylester, Δ^9^-tetrahydrocannabinol, 11-nor-9-carboxy-Δ^9^-tetrahydrocannabinol, amphetamine, methamphetamine, 3,4-methylenedioxymethamphetamine (MDMA), 3,4-methylenedioxyamphetamine (MDA), *N*-methyl-1,3-benzodioxolylbutanamine (MDBD), 3,4-methylenedioxy-*N*-ethylamphetamine (MDEA), ketamine, norketamine, alprazolam, bromazepam, diazepam, nordiazepam, 7-aminoflunitrazepam, lorazepam, amitriptyline, nortriptyline, citalopram, fluoxetine, maprotiline, desmethylmaprotiline, mirtazapine, desmethylmirtazapine, paroxetine, sertraline, desmethylsertraline, venlafaxine, desmethylvenlafaxine, olanzapine, risperidone, 9-hydroxyrisperidone, quetiapine, zolpidem and paracetamol at a concentration of 500 ng/mL each. The specificity study showed that the above substances did not interfere with the determination of the four fentanyl analogues of interest in whole blood samples.

The LODs and LOQs were determined as the sample concentrations corresponding to peak area giving rise to a signal-to-noise (S/N) ratio of at least 3:1 and 10:1, respectively. The LODs were found 0.30 ng/mL for furanylfentanyl and ocfentanil, and 0.15 ng/mL for acetylfentanyl and butyrfentanyl, whilst LOQs were found 1.00 ng/mL for furanylfentanyl and ocfentanil, and 0.50 ng/mL for acetylfentanyl and butyrfentanyl. Representative SIM chromatograms for all analytes at the respective LOQ concentrations are presented in Fig. 1b.

Linearity was evaluated through the calibration curves that were constructed by the method of least-squares with a weighting factor of 1/*x*^2^, and it was expressed by the coefficient of determination (*R*^2^). The calculated calibration curves were found linear (*R*^2^≥ 0.993) within the range of 1.00–100 ng/mL for ocfentanil and furanylfentanyl, and 0.50–50.0 ng/mL for acetylfentanyl and butyrfentanyl. The relative standards of the slope, expressed as percentage, were also calculated and found not greater than 4.9% for all analytes (Table 1).

**Table 1:** Limits of detection **(**LODs), limits of quantification (LOQs) and linearity data of the developed method for the determination of the four fentanils in whole blood

Fentanil	LOD (ng/mL) (S/N ≥ 3/1)	LOQ (ng/mL) (S/N ≥ 10/1)	Concentration range (ng/ml)	% RSD of slopes (*n *= 4)	*R* ^2^
Furanylfentanyl	0.30	1.00	1.0–100	4.1	≥ 0.994
Ocfentanil	0.30	1.00	1.0–100	4.7	≥ 0.993
Acetylfentanyl	0.15	0.50	0.50–50.0	4.6	≥ 0.995
Butyrfentanyl	0.15	0.50	0.50–50.0	4.9	≥ 0.996

*S/N* signal-to-noise ratio, *RSD* relative standard deviation, *R*^2^ coefficient of determination

The recoveries were calculated via analysis and comparison of three replicates at each QC concentration with the respective methanolic standards, because matrix effects were negligible as described above. In all QC levels, the recoveries were ranged from 85.2 to 114% for furanylfentanyl, from 97.1 to 114% for ocfentanil, from 85.0 to 113% for acetylfentanyl and from 97.4 to 116% for butyrfentanyl.

The accuracies of the method were expressed as the percentages of the systematic error (% E_r_) and the precisions as the percentages of relative standard deviation (% RSD). Intraday and interday accuracies were found to be between − 2.7 and 6.0%, for all analytes in all QC levels, and precisions were not greater than 8.0% for all analytes (Table 2).

**Table 2:** Intraday and interday accuracies and precisions of the developed method for the determination of the four fentanils in blood at three QC levels

Fentanil	QC concentration (ng/mL)	Intraday (*n *= 6)			Interday (*n *= 24)		
		Mean concentration ± SD (ng/mL)	Accuracy (% E_r_)	Precision (% RSD)	Mean concentration ± SD (ng/mL)	Accuracy (% E_r_)	Precision (% RSD)
Furanylfentanyl	3.00	2.99 ± 0.20	− 0.33	6.7	2.92 ± 0.22	− 2.7	7.5
	30.0	31.3 ± 1.8	4.3	5.8	30.8 ± 2.0	2.7	6.5
	80.0	83.0 ± 3.1	3.8	3.7	80.3 ± 3.3	0.38	4.1
Ocfentanil	3.00	3.05 ± 0.11	1.7	3.6	2.94 ± 0.15	− 2.0	5.1
	30.0	30.3 ± 1.0	1.0	3.3	30.1 ± 1.7	0.33	5.6
	80.0	78.9 ± 1.8	− 1.4	2.3	80.0 ± 3.1	0.0	3.9
Acetylfentanyl	1.50	1.52 ± 0.05	1.3	3.3	1.50 ± 0.12	0.0	8.0
	15.0	15.4 ± 0.9	2.7	5.8	15.1 ± 1.1	0.67	7.3
	40.0	41.7 ± 1.4	4.2	3.4	40.6 ± 2.1	1.5	5.2
Butyrfentanyl	1.50	1.55 ± 0.06	3.3	3.9	1.49 ± 0.08	− 0.67	5.4
	15.0	15.9 ± 0.8	6.0	5.0	15.2 ± 1.1	1.3	7.2
	40.0	41.2 ± 1.5	3.0	3.6	40.9 ± 1.9	2.2	4.6

*QC* quality control, *SD* standard deviation, *E*_*r*_ error

The stability of the fentanyl analogues in spiked blood samples was assessed after storage of spiked blood samples at room temperature (25 °C) for 1 week, at 4 °C for 24 h, 1 week and 2 weeks and at − 20 °C for 2 weeks and 1 month. Furthermore, frozen spiked blood samples were subjected to three freeze–thaw cycles. The calculated losses for all four fentanyl analogues at all studied conditions are presented in Table 3. All percent values were less than 20%, even storage in whole blood at room temperature (25 °C), showing that they are relatively stable. At − 20 to 4 °C, the percent losses were even lower. To store the whole blood samples for as long as 1 month without any loss, the freezing at − 20 °C is most recommendable.

**Table 3:** Stability of the four fentanils in whole blood under different storage conditions

Storage condition	Percent loss (%)			
	Furanylfentanyl	Ocfentanil	Acetylfentanyl	Butyrfentanyl
1 month (−20 °C)	− 1.0	− 0.53	− 0.80	− 1.5
2 weeks (−20 °C)	− 3.4	− 1.5	− 3.6	− 3.7
2 weeks (4 °C)	− 13	− 7.6	− 13	− 7.8
1 week (4 °C)	− 12	− 7.4	− 13	− 5.2
24 h (4 °C)	− 15	− 12	− 18	− 8.7
1 week (25 °C)	− 14	− 11	− 12	− 11
Three freeze-thaw cycles	− 17	− 9.2	− 18	− 14

### Method application

To our knowledge, there are no seizures of furanylfentanyl, ocfentanil, acetylfentanyl and butyrfentanyl in Greece. Nevertheless, the recent use and prevalence of these analogues around the world has led us to the development of this fit-for-purpose method. The developed method was applied at the Department of Forensic Medicine and Toxicology of National and Kapodistrian University of Athens for the toxicological investigation of 50 forensic cases positive for classic drugs of abuse during the months from September 2017 to May 2018. None of the fentanils was detected in the blood samples. This suggests that the use of these NPS in Greece is, for the time being, limited.

## Conclusions

A quantitative method for the determination of furanylfentanyl and ocfentanil in whole blood, using GC–MS has been developed and validated. This method was also suitable for the determination of acetylfentanyl and butyrfentanyl in the same matrix. The sample pretreatment procedure includes protein precipitation with acetonitrile followed by SPE. To the best of our knowledge, it is the first study to determine furanylfentanyl and ocfentanil by GC–MS. The method presents some considerable advantages as compared to other methods, including liquid chromatography–tandem mass spectrometry, in terms of sensitivity [13, 14, 16, 18, 21, 23, 24], dynamic range [13, 16, 21], and recovery rates. The proposed method can solve already existing analytical problems; it satisfies sensitivity and reliability requirements by using a low cost fundamental equipment, available at common clinical or forensic laboratories, and it can be a useful tool during the toxicological investigation in fentanil related forensic cases.
